# Evaluation of Pupillometry for CYP2D6 Phenotyping in Children Treated with Tramadol

**DOI:** 10.3390/ph16091227

**Published:** 2023-08-30

**Authors:** Frédérique Rodieux, Flavia Storelli, François Curtin, Sergio Manzano, Alain Gervaix, Klara M. Posfay-Barbe, Jules Desmeules, Youssef Daali, Caroline F. Samer

**Affiliations:** 1 Division of Clinical Pharmacology and Toxicology, Department of Anesthesiology, Pharmacology, Intensive Care and Emergency Medicine, Geneva University Hospitals, University of Geneva, Rue Gabrielle-Perret-Gentil 4, 1205 Geneva, Switzerland; 2 Faculty of Medicine, University of Geneva, 1205 Geneva, Switzerland; 3 Division of Pediatric Emergency Medicine, Department of Pediatrics, Gynecology & Obstetrics, Geneva University Hospitals, University of Geneva, 1205 Geneva, Switzerland; 4 Division of General Pediatrics, Department of Pediatrics, Gynecology & Obstetrics, Geneva University Hospitals, University of Geneva, 1205 Geneva, Switzerland; 5 School of Pharmaceutical Sciences, University of Geneva, 1205 Geneva, Switzerland

**Keywords:** children, tramadol, pharmacogenomics, phenotyping, genotyping, CYP2D6

## Abstract

Following the contraindication of codeine use in children, increasing use of tramadol has been observed in pain management protocols. However, tramadol’s pharmacokinetics (PK) and pharmacodynamics are influenced by cytochrome P450 (CYP)2D6 activity, similarly to codeine. Previous studies in adults have demonstrated a correlation between pupillary response and tramadol PK. Our objective was to evaluate pupillometry as a phenotyping method to assess CYP2D6 activity in children treated with tramadol. We included 41 children (mean age 11 years) receiving a first dose of tramadol (2 mg/kg) in the emergency room (ER) as part of their routine care. CYP2D6 phenotyping and genotyping were performed. The concentrations of tramadol and its active metabolite, M1, were measured, and static and dynamic pupillometry was conducted using a handheld pupillometer at the time of tramadol administration and during the ER stay. Pupillometric measurements were obtained for 37 children. Tramadol affected pupillary parameters, with a decrease in pupil diameter in 83.8% of children (*p* = 0.002) (mean decrease 14.1 ± 16.7%) and a decrease in reflex amplitude constriction in 78.4% (*p* = 0.011) (mean decrease 17.7 ± 34.5%) at T150 compared to T0. We were unable to identify a correlation between pupillometry measurements and CYP2D6 activity. Likely confounding factors include light intensity, pain, and stress, making the procedure less feasible in paediatric emergency settings.

## 1. Introduction

Effective and safe analgesic treatments are essential in children, particularly young nonverbal children who are unable to self-report pain and adverse events. Following the contraindication of codeine use in children under 12 years of age and in those undergoing tonsillectomy and/or adenoidectomy, tramadol, although contraindicated in the US, is the only weak opioid still approved for children and used in many countries. Tramadol is the drug of choice for acute nociceptive pain at the Geneva Children’s Hospital, even though its use is made delicate by a large interindividual variability in clinical effect and safety. This variability includes the one common to all opioids, to which is added the variability related to the cytochrome P450 (CYP)2D6 activity.

Tramadol is a prodrug that undergoes liver metabolism through O- and N-demethylation, followed by conjugation. O-demethylation is mediated by CYP2D6, which converts tramadol to its main active metabolite, O-desmethyltramadol (M1) [1,2,3]. Following oral absorption, tramadol and M1 reach peak plasma concentrations after 1–2 h and 3 h, respectively [3,4]. The anti-nociceptive mechanism of action of tramadol is complex, characterised by a dual nature involving both opioid and non-opioid effects. The (+)-tramadol acts as a mu-opioid receptor agonist and inhibits serotonin reuptake, while the (−)-tramadol inhibits noradrenaline reuptake [5,6,7,8]. NA and 5-HT inhibit the transmission of nociceptive impulses by activating α2-adrenergic receptors. Agonism of mu-opioid receptors is mainly ensured by the (+)-enantiomer of M1 [8], which is estimated to be 200 times more potent than the parent compound in binding to mu-opioid receptors and 4–6 times more potent in terms of analgesic effect [2,8,9,10,11,12]. Other possible non-opioid mechanisms of action, such as channel blockade and NMDA receptor antagonism, have been described [13,14,15]. Therefore, similarly to codeine, the analgesic efficacy and safety of tramadol are strongly influenced by CYP2D6 activity [16,17,18].

CYP2D6 activity can be decreased or increased by several pathophysiological factors, such as co-medication, age, comorbidity, and genetic factors [19]. Genetic variations in the *CYP2D6* gene can be used to predict an individual’s CYP2D6 activity, classifying them into one of the four main phenotypes: normal (NM), intermediate (IM), poor (PM), and ultrarapid metabolisers (UM). NM individuals have normal enzymatic activity, IM individuals have reduced enzymatic activity, PM individuals have complete enzyme deficiency, and UM individuals exhibit increased metabolism. The NM and IM phenotypes are the most common in the Caucasian population, accounting for 43–67% and 10–44%, respectively. The IM phenotype represents as much as 50% of the Asian population. PM accounts for 5–10% of the Caucasian population but is rare in other populations. UM phenotypes account for 1–10% of Caucasians but up to 30% of Ethiopians [20,21,22]. Pharmacokinetic (PK) studies have demonstrated that CYP2D6 activity can have a significant impact on the PK, efficacy, and safety of tramadol [16,17,18,23]. PM individuals have impaired tramadol metabolism, leading to lower levels of M1 and a potential reduction in analgesic efficacy and increased risk of serotonin syndrome. In contrast, UM individuals have higher concentrations of M1 and are prone to opioid toxicity [24,25]. Consequently, it could be estimated that around 10% of the population is composed of individuals belonging to the extreme phenotype groups (PM and UM) and may not respond optimally to tramadol treatment, in terms of both effectiveness and adverse effects.

The pre-emptive individual determination of CYP2D6 activity to guide tramadol dosing or the use of alternative medications can significantly improve patient benefit [23]. This is particularly desirable in paediatric patients, who may face challenges in effectively expressing their response to the medication or potential adverse effects due to limited verbal communication abilities. In adults, CYP2D6-activity-guided dosing and therapy, primarily through genotype-guided approaches, have been published for tramadol [26].

CYP activity, including CYP2D6, can be measured by phenotyping and/or predicted by genotyping [27]. CYP450 genotyping is based on DNA analysis and detection of genetic polymorphisms, gene deletion, or copy number variations, and it predicts enzyme activity based on the identified alleles. Although it represents a lifelong trait marker, it does not consider the influence of other factors on CYP450 activity, such as drug–drug interactions, environmental factors, and individual factors [28]. During infancy and childhood, CYP enzymes, including CYP2D6, undergo changes in expression and activity over time—a process known as ontogeny [29,30]. Current data suggest a progressive increase in CYP2D6 activity during the first months and years of life, reaching levels comparable to those seen in adults at one year of age [31,32]. Phenotyping consists of the administration of a validated and selective probe drug metabolised by a specific CYP. Determination of a concentration ratio between the metabolite and the probe (metabolic ratio—MR) in blood or urine makes it possible to define an individual’s metabolic profile [33]. Dextromethorphan (DEM), a cough medication commonly used in children, is considered to be a well-validated probe for assessing CYP2D6 activity by measuring the MR between DEM and its metabolite, dextrorphan (DOR) [33,34,35]. Phenotyping predicts CYP activity at a given timepoint by combining the effects of genetic, environmental, and individual factors, including ontogeny. The non-concordance between the predicted phenotype based on genotype and metaboliser status measured by phenotyping methods is termed “phenoconversion” [36,37].

Routine implementation of the methods described above is often challenging in children due to sampling procedures, the need for probe drug administration, concerns about unwanted adverse effects of the probe drug, and the time required to obtain results. Given these considerations, alternative methods are needed. In adults, pupillary response has been correlated with tramadol PK [17,38,39,40], suggesting the possibility of rapid phenotyping of CYP2D6 by measuring pupillary response. Pupillary response, pupillary diameter (PD), and pupillary light reflex (PLR) are regulated by a complex interaction of several intrinsic and extrinsic factors, including light intensity and visual accommodation [41], the autonomic nervous system [42,43], emotional state [44], old age in adults (but not in children) [45,46,47], pain [47,48], eye and brain diseases, iris colour [49], and various drugs, including opioids [43,50,51,52]. The pupillary response is primarily influenced by the intensity of the light reaching the retina and accommodation for near vision [41]. In bright light, the pupil contracts to reduce light’s entry, while in dim light the pupil dilates to improve visual sensitivity [45]. The sympathetic system primarily controls the contraction of the radialis muscle, resulting in pupil dilatation, while the parasympathetic system regulates the pupillary sphincter, resulting in pupil constriction [53,54]. Opioids regulate PD by binding to mu receptors, stimulating the parasympathetic Edinger–Westphal nucleus of the common ocular motor nerve (III) [42], which decreases sympathetic brake and leads to pupil constriction (miosis) [55]. Studies have clearly reported a relationship between opioid dosing or plasma concentrations and their effects on PD [47,52,56], and PD has been used to assess responses to opioid medications in adults [38,39,50,57]. The effects of opioids on PLR parameters are less well characterised, and the results are inconclusive [38,39,47,51,52,58,59]. Tramadol has a confirmed miotic effect, although evidence suggests that it may be weaker and delayed compared with full opioid mu-agonists [39,54]. The effect of tramadol on the pupil seems to be complex due to the interaction and antagonistic effects of its opioid and monoaminergic properties, i.e., constriction induced by the mu component and dilatation induced by noradrenaline reuptake blockade and indirect stimulation of alpha-adrenergic receptors by the parent drug [5,60]. The final impact of this complex interaction on the pupil is not yet fully understood. Current data in adults suggest a greater influence of M1 than tramadol [39,54], with variation in this interaction over time due to the distinct kinetics of tramadol and M1, and consequent dynamic changes in the M1/tramadol ratio over time [39]. There are no specific data for children.

Studies have shown that tramadol has a dose- and CYP2D6-activity-dependent effect on the pupillary response, leading to a decrease in resting PD [38,39,52], as well as a decrease in the amplitude and velocity of constriction and an increase in latency [39]. In most cases, no significant effect was observed in volunteers receiving small doses of tramadol or in patients classified as CYP2D6 PM [38,39]. Specific data for children are not currently available.

Pupillometry using automated handheld pupillometers is a non-invasive, safe, rapid, and easy method for measuring a range of objective pupillary variables, including size, constriction velocity, and dilation velocity.

Based on the hypothesis of a positive relationship between the amount of M1 formed and the intensity of miosis after the administration of tramadol [39], the aim of this study was to evaluate pupillometry as an alternative phenotyping method to identify CYP2D6 phenotype extremes (i.e., UM and PM) in children treated with tramadol for pain management in the emergency room (ER), i.e., to assess whether a correlation was observed between pupillometry parameters and CYP2D6 activity determined by phenotyping using DOR/DEM MR, M1/tramadol MR, or M1 concentration in the blood after tramadol administration. The tolerability and feasibility of pupillometry (infrared NeurOptics NPi-200^®^ Pupillometer, San Clemente, CA, USA) in children were also assessed. The secondary outcomes included the assessment of the correlation between DOR/DEM MR and M1/tramadol MR, as well as the concordance between CYP2D6 activity predicted by the genetic activity score (AS) and the DOR/DEM and M1/tramadol MR.

## 2. Results

### 2.1. Population Characteristics and Demographics

Between March 2017 and June 2022, a total of 50 children were enrolled in the study. As the study should not interfere with the child’s routine care management (i.e., not keeping a child in the ER longer than expected) and the running of the ER (i.e., no occupation of a consultation room longer than planned), nine children had to be excluded. The reasons for exclusion were the end of care and desire to go home before the study’s completion (n = 7), and the need to introduce a treatment interacting with the study (morphine n = 1 and fentanyl n = 1).

In the end, we included 41 children (26 boys and 15 girls). The characteristics of the final study population are described in Table 1. The mean age and weight were 11 years (range 3–15 years) and 48.1 kg (range 17–103 kg), respectively. The population was ethnically diverse, with the majority being Caucasian (n = 22). The phenotypes predicted on the basis of the alleles identified (genotype) are presented in Table 2. Our population was composed of 63.4% NM, 29.3% IM, 4.9% UM, and 2.4% PM, which corresponds to the expected distribution of CYP2D6 phenotypes in a cosmopolitan urban population [61]. The number of children in the extreme phenotype groups (PM and UM) was less than 10%.

The mean dose of tramadol (Tramal^®^ dosing pump: 8 pushes = 1 mL = 100 mg tramadol chlorhydrate, commercialised in Switzerland, Grünenthal) was 1.66 mg/kg (SD 0.35; range 0.64–2.22). As per protocol, the oral sub-therapeutic dose of DEM used for phenotyping (Bexine syrup^®^: 1 mL = 2.5 mg dextromethorphan, commercialised in Switzerland, Spirig HealthCare AG) was 0.15 mg/kg for all children, except for one child who mistakenly received a therapeutic dose of 0.375 mg/kg.

**Figure 1:**
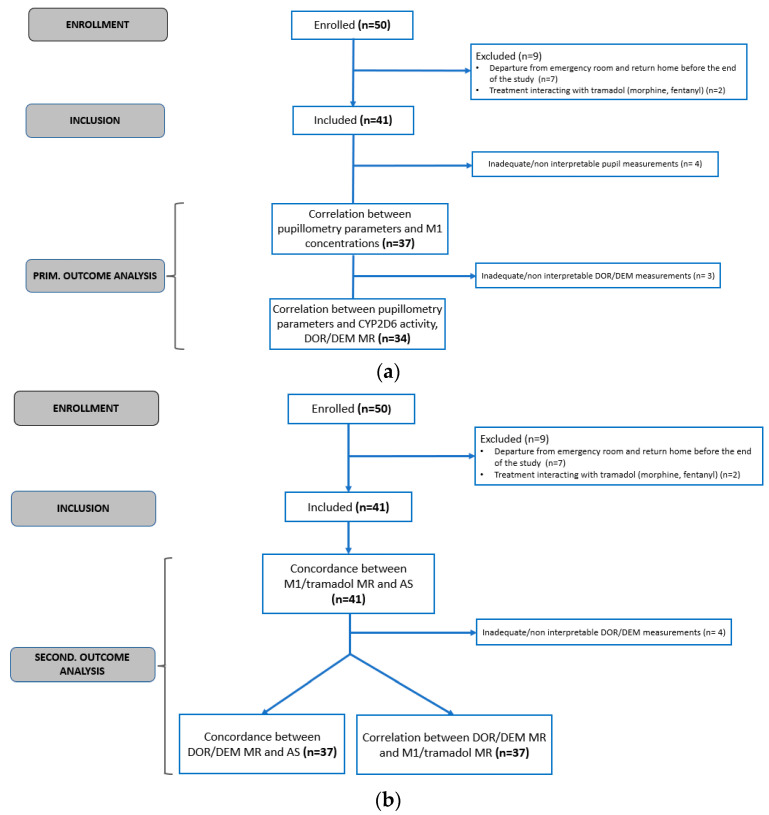
Study flowcharts for primary (**a**) and secondary (**b**) outcomes.

Of the 41 children, only 37 had pupillometry measurements that could be interpreted for the primary outcome (Figure 1) (i.e., a minimum of three timepoints, including the first measurement and a final measurement at least 90 min after tramadol administration).

### 2.2. Correlation between Pupillometry Parameters and CYP2D6 Activity

Among the children with pupillometry measurements, a total of 37 children were phenotyped by DOR and DEM measurements, as well as M1 and tramadol concentration measurements. Of these, 34 children had interpretable DOR/DEM MR, and all 37 children had measurements of M1 and tramadol concentrations (Figure 1a).

In 18 children, the first pupillometry measurement was conducted before or at the same time as tramadol administration. For the remaining participants, the first measurement was taken on average 8 min (SD 6; range 1–22) after tramadol administration. The timing and frequency of pupillary response measurements varied significantly between children, with an average of 5.5 measurements (ranging from 4 to 8) per child. On average, the last measurement was taken 156 min after tramadol administration (SD 45). The time intervals between the administration of tramadol and DEM and blood sampling were 150 (range 109–280) and 126 (range 90–265) min, respectively. In four children, the results of the DEM measurement were considered to be falsely elevated and uninterpretable, with DEM values ranging from 15 to 25 times the expected concentrations for the given dose. We assumed that analytical interference had occurred. These children were removed from all data analyses involving the DOR/DEM MR.

Light intensity varied significantly between different participants and within the same child throughout the study. The lowest light intensity recorded was 29 lux, while the highest was 760 lux, with an average of 205 lux. Compared with the initial measurement, none of the subsequent measurements showed a variation in light intensity greater than a 50% increase or decrease. On average, the percentage change in light intensity compared with the baseline measurement for the same children was 6.5% (SD 24.7).

Given the PK of tramadol and its metabolites, whose Cmax is reached after approximately 1–2 h and 3 h, respectively, we chose to examine the correlation between the DOR/DEM MR and pupil measurements taken as close as possible to 150 min (referred to as T150). On average, T150 measurements were taken 132 min (SD 28) after tramadol administration.

Following tramadol administration, a miotic reaction, characterised by a decrease in initial resting PD (restPD), was observed in 83.8% of the children (*p* = 0.002). An increase in restPD was observed in six children (16.2%). The extent of the miotic reaction varied between individuals, with a mean decrease in restPD of 0.81mm (SD 0.90) and 14.1% (SD 16.7) in the absolute value and percentage variation, respectively. Multiple regression analyses were performed to predict the variation in restPD between T0 and T150 as a function of the CYP2D6 activity. We did not find any relation between the degree of miosis between T0 and T150 and the CYP2D6 activity estimated with DOR/DEM MR (*p* = 0.308), M1/tramadol MR, or the M1 concentration (*p* = 0.243) (Table 3). A search of the confounding variable revealed a significant positive association between the degree of miosis and the mean lux during the study (*p* = 0.003).

Among the PLR parameters, a decrease in the reflex amplitude constriction (RA)—an indicator of loss of pupillary reactivity—between T0 and T150 was observed in 78.4% of the children (*p* = 0.011), with a mean decrease in RA of 0.45 mm (SD 0.56) and 17.7% (SD 34.5) in the absolute value and percentage variation, respectively. All other variables showed large interindividual variability, with no significant changes after tramadol administration. There was a non-significant inverse relationship between M1 concentration and the difference in RA between T0 and T150 (*p* = 0.293) (Table 4).

#### Feasibility and Safety

No adverse events, discomfort, or injuries occurred during the study because of the pupillometer, tramadol, or DEM administration. The pupillometer was easy to use. No defects occurred. No pupillometry measurements were refused during the study.

### 2.3. Correlation between DOR/DEM MR and AS 

Thirty-seven children were simultaneously genotyped and phenotyped using DEM and subjected to measurements of tramadol and M1 concentrations (Figure 1b).

**Figure 2:**
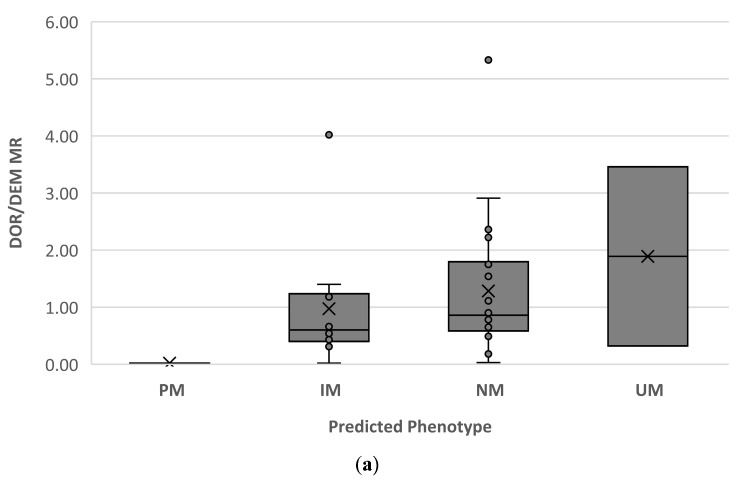

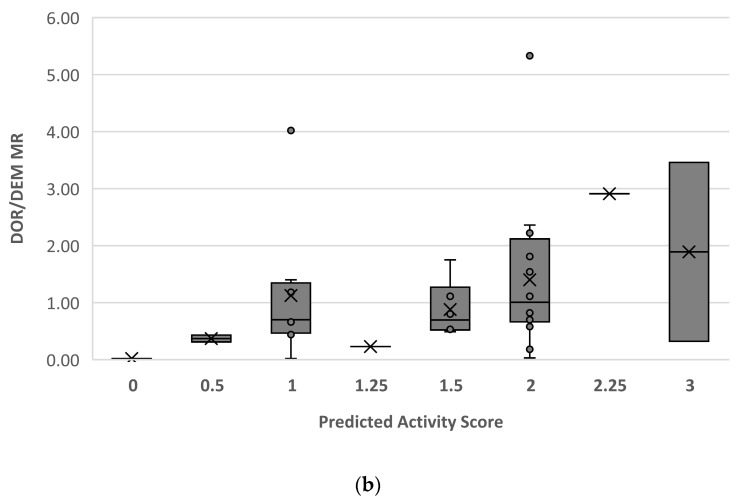
Distribution of dextrorphan-to-dextromethorphan metabolic ratio (DOR/DEM MR) for each predicted phenotype (**a**) and each assigned activity score (AS) based on the genotype (**b**), according to Caudle et al. [62]. DOR/DEM, dextrorphan to dextromethorphan; MR, metabolic ratio; PM, poor metaboliser; IM, intermediate metaboliser; NM, normal metaboliser; UM, ultrarapid metaboliser. Boxes indicate the interquartile ranges; dots represent observations, and crosses denote mean values.

Figure 2 shows the distribution of DOR/DEM MR for each predicted phenotype (Figure 2a) and each assigned AS based on the genotype (Figure 2b).

In 56.8% of cases (21/37), concordance was observed between the phenotype predicted by genotyping and the phenotype measured with DOR/DEM MR. Details of concordance and discordances are presented in Table 5. Of the 16 cases with a discordance between genotype and phenotype, 75% (12/16) had a measured activity lower than predicted by the genotype (UM → NM/IM, n = 2; NM → IM, n = 8; NM → PM, n = 1; IM → PM, n = 1). The lack of concordance was not limited to young children but was observed in children of all ages (from 6 to 15 years old). The non-concordance could not be explained by comedication-induced phenoconversion in any of these cases.

The relationship between DOR/DEM MR and M1/tramadol MR was analysed by Pearson’s test. As expected, the correlation was significantly positive (r = 0.513, *p* = 0.001).

**Figure 3:**
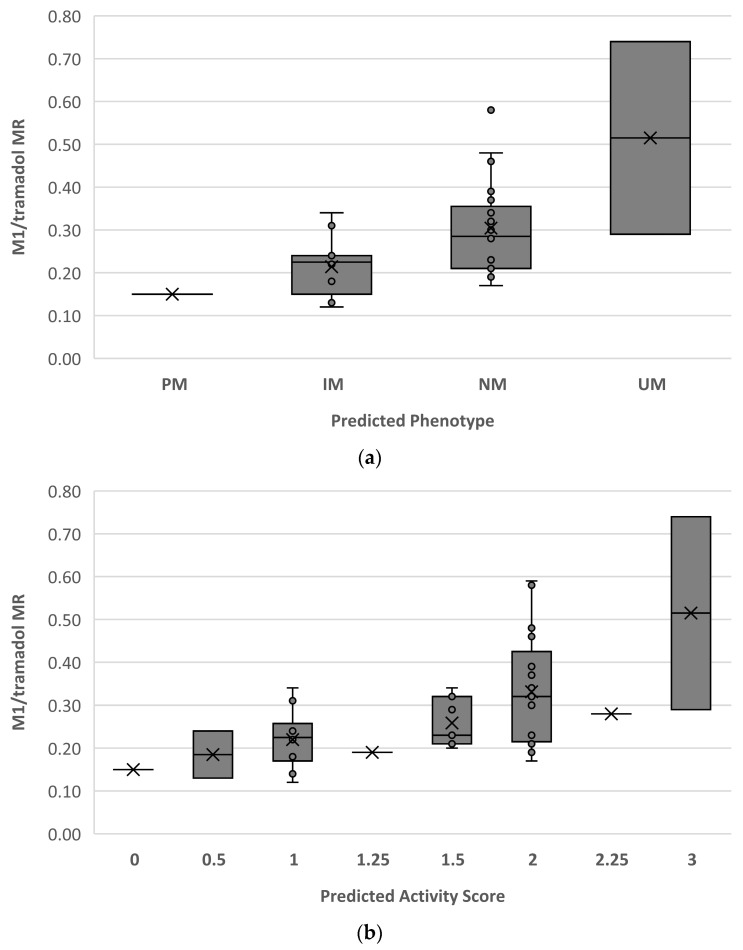
Distribution of O-desmethyltramadol (M1)/tramadol metabolic ratio (MR) for each predicted phenotype (**a**) and each assigned activity score (AS) based on the genotype (**b**), according to Caudle et al. [62]. M1, O-desmethyltramadol; MR, metabolic ratio; PM, poor metaboliser; IM, intermediate metaboliser; NM, normal metaboliser; UM, ultrarapid metaboliser. Boxes indicate the interquartile ranges; dots represent observations, and crosses denote mean values.

Figure 3 shows the distribution of M1/tramadol MR for each predicted phenotype (Figure 3a) and each assigned AS according to genotype (Figure 3b).

## 3. Discussion

In our study, we expected changes in pupillary response, PD, and PLR after tramadol administration, as well as changes related to the amount of M1 metabolite and CYP2D6 activity. No correlation was observed between CYP2D6 activity (determined by DOR/DEM MR) or the concentration of M1 and any pupillary parameter, static or dynamic. Consistent with previous studies, we observed decreases in restPD and RA after tramadol administration. In addition, although not statistically significant, a negative relationship was observed between the concentration of M1 and RA, in agreement with the observations of Fliegert et al. [39]. However, a number of limitations and confounding factors need to be considered when interpreting these results, such as the lack of standardised conditions for pupillometry measurements, particularly with regard to the influence of light. Other factors known to affect pupillary response, such as iris colours and accommodation, but also pain intensity, fear, and emotion triggered by an ER visit, should also be taken into account. The literature suggests that DEM has an ability to antagonise NMDA receptors, in addition to inhibiting noradrenaline reuptake and being an alpha-agonist [63,64]. Therefore, although a significant effect on the pupil is not expected at a dose of 0.15 mg/kg, the potential mydriatic influence of DEM on pupillary response cannot be completely ruled out. The short duration of our study, designed not to disrupt the ER’s operation and routine care management, along with the small patient sample, may have limited our ability to observe significant effects. The possibility of an insufficient dose cannot be ruled out. Lastly, the study did not take into account the variability in opioid response due to functional polymorphisms of the mu-opioid receptor.

Regarding secondary outcomes, this study showed a good relationship between the DOR/DEM MR and the M1/tramadol MR. Both MRs showed a satisfactory distribution for each phenotype predicted as a function of genotype (Figure 2 and Figure 3). However, discordances between the phenotype determined by the DOR/DEM MR and the genotype were observed. Our study showed a concordance rate of 58%, which is consistent with previous findings in children [65]. In this study, the non-concordance could not be explained by comedication-induced phenoconversion. In most cases of non-concordance, with measured activity below predicted activity, a role of ontogeny would have been invoked, but the observation of this pattern across different ages, including adolescents, suggests that CYP2D6 ontogeny may not play a significant role. The limited sensitivity of genotyping tests, as well as default calls and unidentified genetic variations or environmental factors may have contributed to some discordant cases [66]. The challenges associated with the broad and currently unvalidated cutoff values for CYP2D6 phenotype classification in children should be considered. Further studies on larger samples of children are needed to confirm our findings and, if necessary, reassess the cutoff values for children.

Finally, our study highlights the difficulty of recruiting paediatric patients and conducting studies in the ER setting. Low enrolment is a common phenomenon in paediatric research, and previous studies have already identified a number of challenges to participation in paediatric clinical trials, including difficulties in obtaining parental consent, logistical challenges, uncomfortable study procedures, and clinical overload [67,68,69,70,71]. According to one survey, 31% of paediatric clinical studies are discontinued due to low enrolment, and one-third of trials do not reach 80% of target enrolment before closure [71]. Successfully conducting a study in an ER requires not only accommodating the demands of routine patient care, but also adapting to the responsibilities and workload of caregivers.

Our study encountered most of the above obstacles, including difficulties faced by our primary nurses in identifying potential participants and notifying the research team, obtaining parental consent in the ER, adding an unnecessary blood test to a child’s care, or the unavailability of study staff due to their heavy workload. Complexity stems from the difficulty of standardising the study environment, i.e., taking measurements and samples at specific and predetermined times, but also maintaining a constant light intensity throughout the study, due to frequent patient displacement (between the waiting room, the examination room, the X-ray rooms, etc.). Additionally, the decrease in tramadol prescriptions in favour of fentanyl, along with the impact of the global COVID-19 pandemic on paediatric ER visits, further affected participants’ recruitment. Consequently, our study was stopped prematurely, and the small number of children included—especially in the extreme phenotype groups (PM and UM)—may have influenced our results and prevented evidence-based conclusions.

We can conclude that the pupillometer is safe and well tolerated by children and simple to use by caregivers. However, despite encouraging data in adults suggesting the possibility of rapid determination of CYP2D6 activity in patients treated with tramadol, our study does not currently support the use of the pupillometer as a reliable and easy non-invasive method for phenotyping to personalise pain management in paediatric ER settings. However, it suggests that with better standardisation, such as control of light intensity, minimisation of accommodation, and repletion of measurements, pupillometry could become a promising tool, but in an environment subject to fewer constraints than the ER.

Furthermore, our findings also highlight that low-dose DEM did not cause any safety events and can be safely used in children for CYP2D6 phenotyping. Additionally, measuring the M1/tramadol MR after the first dose of tramadol treatment could be used as a phenotyping method, eliminating the need to administer a probe substrate. Future studies are needed to confirm this hypothesis and to define the optimal time for measuring M1/tramadol MR and cutoff values. Further studies are also needed to establish validated cutoff values for DOR/DEM MR in the paediatric population.

## 4. Material and Methods

### 4.1. Trial Design

We conducted a single-centre, open-label interventional study at the Geneva Children’s Hospital (Hôpitaux Universitaires de Genève, HUG) after approval from the local ethics committee and international registration (ClinicalTrials.gov Identifier: NCT03052218), and in accordance with the Declaration of Helsinki guidelines, between March 2017 and June 2022. Infants and children aged between 1 and 15 years, weighing more than 10 kg, with no neurological or ophthalmological deficits, and who received their first dose of oral tramadol for pain management in the ER as part of their usual care, were eligible to participate in the study. The tramadol dosage administered to all participants was based on the recommendations of the Swiss agency for therapeutic products, Swissmedic, and the current guidelines at the Geneva Children’s Hospital, which are 2 mg/kg (maximum 100 mg) (Tramal^®^ dosing pump: 8 pushes = 1 mL = 100 mg tramadol chlorhydrate, commercialised in Switzerland, Grünenthal).

The main exclusion criteria for participation included known kidney or liver disease and concomitant treatment with CYP2D6 inhibitors and CYP3A inhibitors/inducers [27], and/or with a drug known to PD. Participants with documented previous adverse events (AE) to tramadol or DEM were also excluded from the study.

### 4.2. Intervention

After obtaining informed consent from the parents or legal guardians of eligible children (and from the children if aged 14 years or older), we collected anthropometric parameters, vital signs, and medical history, including information on any concomitant treatments. Physical examination was part of routine care. The first measurement of PD was performed using a pupillometer before or, if this was not possible due to childcare management, within 20 min of tramadol administration. This initial measurement was used as a baseline. Subsequent measurements were taken once or twice per hour during the children’s stay in the ER. A minimum of three timepoints were required, including the first measurement and a final measurement at least 90 min after tramadol administration. As soon as possible after inclusion, we administered a single oral dose of DEM (0.15 mg/kg. max 10 mg) (Bexine syrup^®^: 1 mL = 2.5 mg dextromethorphan, commercialised in Switzerland, Spirig HealthCare AG) to the participant for CYP2D6 phenotyping. At least 90 min after DEM administration, a blood sample was taken to measure DEM, its metabolite DOR, tramadol, and its active metabolite M1. Additionally, genotyping of the *CYP2D6* gene was performed.

### 4.3. Phenotyping

CYP2D6 phenotyping was performed using DEM in accordance with local practice at Geneva University Hospitals. To ensure that the administration of this probe was sub-therapeutic, but also to take into account the detection limits of our analytical method (especially considering the possibility of including young children weighing as little as 10 kg, in accordance with our protocol), a single oral dose of DEM syrup 0.15 mg/kg was administered.

Two hours later (with an acceptable delay of 1 to 6 h due to the demonstrated stability of DOR/DEM MR over time [63]), capillary or venous whole blood was collected [64]. The capillary route was used when the child had no peripheral venous access. Venous blood was used when there was functional venous access or concomitant blood sampling. DEM and DOR, tramadol, and its M1 metabolite were measured by a validated liquid chromatography–tandem mass spectrometry quantification method [34,35,72]. Children were classified as CYP2D6 PM, IM, NM, or UM based on their DOR/DEM MR and validated adult cutoff values [35] (Table S1).

### 4.4. Genotyping

DNA was extracted from the blood sample. The institutional laboratory of molecular oncology and pharmacogenomics performed CYP2D6 genotyping by Sanger sequencing and TaqMan copy number analysis. Clinical Pharmacogenetic Implementation Consortium (CPIC) guidelines were used to calculate AS from diplotype [26]. Children were classified into PM, IM, NM, and UM based on their AS range, in accordance with current CPIC recommendations [62] (Table 6). In this manuscript, we use the term predicted phenotype to refer to this genotype-predicted group assignment.

### 4.5. Pupillometry

Static and dynamic pupillometry was performed using a commercial infrared NeurOptics NPi-200^®^ Pupillometer (NeurOptics, San Clemente, CA, USA) from Unimedtech Switzerland. This belongs to the category of class I medical devices and carries a CE marking the NeurOptics NPi^®^. A pupillometer is a non-invasive, handheld, cordless, and easy-to-use quantitative infrared device used in adults as well in children [73]. It was used in this trial according to the manufacturer’s instructions. It provides objective static restPD (mm) and reactivity data 3 s after a brief light stimulus—termed the PLR—independent of the examiner. The PLR waveform is measured and the pupillometer automatically calculates and reports latency (s), average and maximum pupil constriction velocity (mm/s), minimum PD (minPD) at the peak of constriction (mm), percent (%) change (CH) in PD (restPD vs. minPD) and dilatation velocity (DV) (mm/s), and the Neurological Pupil Index (NPi) (Table 7). The NPi is a number calculated by a proprietary algorithm that takes all measured variables in the PLR to give a composite score and compares it to known normal observations [74]. We defined the RA as the absolute difference between the restPD and the minPD. The PD and reactivity measurements did not require any contact with the patient’s eyes or skin. If the pupillometer failed to track the pupil because of eye blinks, eyelid closure, or eye movement, the measurement was discarded and repeated. The children were instructed to focus on a distant object to stabilise accommodation, but in order not to add additional stress to the child, the subject’s other eye was not covered to block out ambient light. The ambient light level in the room was measured at the same time as each measurement in order to conduct the study in a room with an average light intensity between 50 and 500 lux; these light intensity levels correspond to common light levels indoors for normal activities. The stability of light intensity during the study was desired. We arbitrarily decided to measure the left eye systematically, as the average between the two eyes could not calculated because some measurements were not taken at exactly the same time.

### 4.6. Statistical Analysis

We hypothesised that the CYP2D6 phenotype (expressed as DOR/DEM MR) would be correlated with the initial velocity of pupil size reduction after tramadol uptake, and that the correlation coefficient would lie within a 95% confidence interval between 0.68 and 0.88. Expecting a correlation coefficient of 0.8, 53 participants were required to obtain a 95% confidence interval from 0.68 to 0.88. In addition, considering the frequencies of CYP2D6 UM and PM phenotypes—expected to be around 5 to 10% in the local population of Geneva, according to previous studies by our group—this number would have allowed for the observation of a few cases of extreme phenotype groups in the study sample.

We used descriptive statistics to describe our data. Categorical variables were described using frequency tables (n, % or range), whereas continuous variables were described using means (±standard deviation).

Differences in percentages were analysed with the chi-squared test. Overall comparisons of changes in PD and PLR parameters relative to baseline were performed. Multiple regression was used to determine the relationships between the different pupil parameters, M1 metabolite concentration, and DOR/DEM with lux. Pearson’s correlation was used to determine the relationship between the DOR/DEM MR and the M1/tramadol MR.

Data analysis was performed using the SPSS^®^ software package, version 25 (IBM corporation, Armonk, NY, USA). A *p-*value of less than 0.05 was considered statistically significant.

### 4.7. Assessment of Adverse Events

All clinical AEs reported spontaneously by the children and/or their parents in response to an open question from the study personnel were recorded. The incidence, nature, severity, and outcome of the AEs were systematically recorded. All AEs were monitored until remission.

## Figures and Tables

**Table 1:** Children’s demographics. Mean, (range).

Number of individuals (n total = 41)	
- Female	15
- Male	26
Age (years)	11 (3–15)
Weight (kg)	48.1 (17–103)
Source of pain	
- Traumatic	26
- Non-traumatic	15

**Table 2:** Cytochrome P450 *2D6* diplotype, assigned AS, and predicted metabolic phenotype.

Predicted Metabolic Phenotype Numbers of Individual (%)	PM 1 (2.4)	IM 12 (29.3)		NM 26 (63.4)				UM 2 (4.9)
Assigned AS Numbers of individual (%)	0 1 (2.4)	0.5 2 (4.9)	1.0 10 (24.4)	1.25 1 (2.4)	1.5 7 (17.1)	2.0 17 (41.5)	2.25 1 (2.4)	3 2 (4.9)
Genotype; *CYP2D6* diplotype	*4/*4	*4/*41; *10/*10;	*1/*4; *2/*6; *1/*6; *1/*4 × N; *2/*5; *4j/*35	*1/*10	*1/*41 *1/*17 *2/*41 *2/*9 *1/*9	*1/*1 *1/*35 *1/*41 × N *1/*2 *2/*2	*1 × 2/*10	*2/*35 × N *2/*2 × N

CYP, cytochrome P450; PM, poor metaboliser; IM, intermediate metaboliser; NM, normal metaboliser; UM, ultrarapid metaboliser; AS, activity score; × N, 2 or more gene copies.

**Table 3:** Multiple linear regression associations of resting pupil diameter variation (mm) between T0 and T150 with dextrorphan-to-dextromethorphan metabolic ratio and mean lux variables (6a), M1-to-tramadol metabolic ratio and mean lux variables (6b), and O-desmethyltramadol concentration and mean lux variables (6c).

		Dependent Variable: Delta RestPD between T0 and T150 (mm)			
a.	Independent Variables	B	95% CI	*p*	
	DOR/DEM MR	0.127	[−0.123, 0.378]	0.308	(n = 34) (R^2^ = 0.252, *p* = 0.011 *).
	Mean lux	0.003	[0.001, 0.005]	0.003 **	
b.	Independent variables	B	95% CI	*p*	
	M1/tramadol MR	−0.033	[−2.223, 2.160]	0.976	(n = 37) (R^2^ = 0.232, *p* = 0.011 *).
	Mean lux	0.003	[0.001, 0.004]	0.003 **	
c.	Independent variables	B	95% CI	*p*	
	M1 concentration (LN)	−0.243	[−0.659, 0.172]	0.243	(n = 37) (R^2^ = 0.263, *p* = 0.006 **).
	Mean lux	0.003	[0.001, 0.004]	0.003 **	

RestPD, resting pupil diameter; mm, millimetre; B, coefficient beta; CI, confidence interval; DOR/DEM, dextrorphan to dextromethorphan; MR, metabolic ratio; M1, O-desmethyltramadol; LN, natural logarithm; * *p* < 0.05; ** *p* < 0.01.

**Table 4:** Multiple linear regression associations of reflex amplitude constriction variation between T0 and T150 (mm) with M1 concentration and mean lux variables.

Dependent Variable Delta RA between T0 and T150 (mm).				
Variable	B	95% CI	*p*	
M1 concentration (LN)	−0.140	[−0.407, 0.127]	0.293	
Mean lux	0.001	[0.000, 0.003]	0.007 **	(n = 37) (R^2^ = 0.229, *p* = 0.012 *).

RA, reflex amplitude constriction; mm, millimetre; B, coefficient beta; CI, confidence interval; M1, O-desmethyltramadol; LN, natural logarithm; * *p* < 0.05; ** *p* < 0.01.

**Table 5:** Concordance between the predicted and measured phenotype, given as n (%).

Predicted Phenotype Based on Genotype		Measured Phenotype (DOR/DEM MR)			
		PM (%) ^a^	IM (%) ^a^	NM (%) ^a^	UM (%) ^a^
PM	1	1 (100%)	0	0	0
IM	10	1	6 (60%)	3	0
NM	24	1	8	14 (58%)	1
UM	2	0	1	1	0 (0%)

^a^ Percent concordance of measured phenotype with predicted phenotype based on genotype. DOR/DEM, dextrorphan to dextromethorphan; MR, metabolic ratio; PM, poor metaboliser; IM, intermediate metaboliser; NM, normal metaboliser; UM, ultrarapid metaboliser.

**Table 6:** Assignment of predicted CYP2D6 phenotypes based on diplotypes, [26].

Predicted Phenotype	Activity Score Range	Activity Score/Genotypes	Considered Diplotypes
CYP2D6 ultrarapid metaboliser (UM)	>2.25	>2.25	*1/*2 × N, *2/*35 × N
CYP2D6 normal metaboliser (NM)	1.25 ≤ × ≤ 2.25	1.25 1.5 1.75 2.0 2.25	*1/*10 *1/*41, *1/*17, *2/*41, *2/*9 - *1/*1, *1/*35, *1/*41 × N, *1/*2, *2/*2 *1 × 2/*10
CYP2D6 intermediate metaboliser (IM)	0 < × < 1.25	0.25 0.5 0.75 1	- *4/*41, *10/*10 - *1/*4, *2/*6, *1/*6, *1/*4 × N, *2/*5, *4j/*35
CYP2D6 poor metaboliser (PM)	0	0	*4/*4

**Table 7:** Pupillary measurement parameters.

Static	Initial Resting Pupil Diameter = RestPD	Maximum Pupil Size before Constriction
Dynamic	CH = % Change	% of change = minPD/restPD as a %
	minPD = minimum pupil diameter	PD at peak of the constriction
	LAT = latency of constriction	Time of onset of constriction following initiation of the light stimulus
	CV = average constriction velocity	Average of how fast the PD is constricting, measured in millimetres per second
	MCV = maximum constriction velocity	Maximum velocity of pupil constriction of the PD responding to the flash of light, measured in millimetres per second
	DV = dilation velocity	The average pupillary velocity when, after having reached the peak of constriction, the pupil tends to recover and to dilate back to the initial size, measured in millimetres per second
	NPi^®^ = Neurological Pupil Index™	Numerical expression of pupil reactivity

## Data Availability

The data that support the findings of this study are available upon request from the corresponding author.

## Supplementary Materials

The following supporting information can be downloaded at: https://www.mdpi.com/article/10.3390/ph16091227/s1, Table S1: Assignment of predicted cytochrome P450 (CYP)2D6 phenotypes based on dextrorphan-to-dextromethorphan metabolite ratio (DOR/DEM MR) cutoff values [35].

## Abbreviations

CYP	Cytochrome P450
M1	O-desmethyltramadol
MR	Metabolic ratio
DEM	Dextromethorphan
DOR	Dextrorphan
PD	Pupil diameter
ER	Emergency room
NM	Normal metaboliser
PM	Poor metaboliser
IM	Intermediate metaboliser
UM	Ultrarapid metaboliser
CPIC	Clinical Pharmacogenetic Implementation Consortium
AS	Activity score
restPD	Initial resting PD
PLR	Pupillary light reflex
minPD	Minimum PD at peak of the constriction
CH	% Change
LAT	Latency of constriction
CV	Average constriction velocity
MCV	Maximum constriction velocity
DV	Dilation velocity
NPi	Neurological Pupil Index™
RA	Reflex amplitude constriction
AE	Adverse event
