# Supplementary material for: Evaluation of Pupillometry for CYP2D6 Phenotyping in Children Treated with Tramadol
Source: Pharmaceuticals (Basel). 2023 Aug 30;16(9):1227. doi: 10.3390/ph16091227 (PMC10537526; doi:10.3390/ph16091227)
Supplement: Supplementary file 1 [file pharmaceuticals-16-01227-s001.zip › pharmaceuticals-2516135-SI.pdf]

Supplementary material

Table S1: Assignment of predicted Cytochrome P450 (CYP)2D6 phenotypes based on dextrorphan to dextromethorphan metabolite ratio (DOR/DEM MR) cut-off values, [35].

**Poor metabolizer (PM)** (Mean MR ± SD)	**Intermediate metabolizer IM** (Mean MR ± SD)	**Normal metabolizer NM** (Mean MR ± SD)	**Ultrarapid metabolizer UM** (Mean MR ± SD)
0.05 ± 0.02	0.46 ± 0.41	2.41 ± 1.79	NA

SD, standard deviation
